# Bgee in 2024: focus on curated single-cell RNA-seq datasets, and query tools

**DOI:** 10.1093/nar/gkae1118

**Published:** 2024-12-05

**Authors:** Frederic B Bastian, Alessandro Brandulas Cammarata, Sara Carsanaro, Harald Detering, Wan-Ting Huang, Sagane Joye, Anne Niknejad, Marion Nyamari, Tarcisio Mendes de Farias, Sébastien Moretti, Marianna Tzivanopoulou, Julien Wollbrett, Marc Robinson-Rechavi

**Affiliations:** Evolutionary Bioinformatics, SIB Swiss Institute of Bioinformatics, Quartier Sorge, Bâtiment Amphipôle, Lausanne, 1015, Switzerland; Department of Ecology and Evolution, University of Lausanne, Quartier Sorge, Bâtiment Biophore, Lausanne, 1015, Switzerland; Evolutionary Bioinformatics, SIB Swiss Institute of Bioinformatics, Quartier Sorge, Bâtiment Amphipôle, Lausanne, 1015, Switzerland; Department of Ecology and Evolution, University of Lausanne, Quartier Sorge, Bâtiment Biophore, Lausanne, 1015, Switzerland; Evolutionary Bioinformatics, SIB Swiss Institute of Bioinformatics, Quartier Sorge, Bâtiment Amphipôle, Lausanne, 1015, Switzerland; Department of Ecology and Evolution, University of Lausanne, Quartier Sorge, Bâtiment Biophore, Lausanne, 1015, Switzerland; Evolutionary Bioinformatics, SIB Swiss Institute of Bioinformatics, Quartier Sorge, Bâtiment Amphipôle, Lausanne, 1015, Switzerland; Department of Ecology and Evolution, University of Lausanne, Quartier Sorge, Bâtiment Biophore, Lausanne, 1015, Switzerland; Evolutionary Bioinformatics, SIB Swiss Institute of Bioinformatics, Quartier Sorge, Bâtiment Amphipôle, Lausanne, 1015, Switzerland; Department of Ecology and Evolution, University of Lausanne, Quartier Sorge, Bâtiment Biophore, Lausanne, 1015, Switzerland; Evolutionary Bioinformatics, SIB Swiss Institute of Bioinformatics, Quartier Sorge, Bâtiment Amphipôle, Lausanne, 1015, Switzerland; Department of Ecology and Evolution, University of Lausanne, Quartier Sorge, Bâtiment Biophore, Lausanne, 1015, Switzerland; Evolutionary Bioinformatics, SIB Swiss Institute of Bioinformatics, Quartier Sorge, Bâtiment Amphipôle, Lausanne, 1015, Switzerland; Department of Ecology and Evolution, University of Lausanne, Quartier Sorge, Bâtiment Biophore, Lausanne, 1015, Switzerland; Evolutionary Bioinformatics, SIB Swiss Institute of Bioinformatics, Quartier Sorge, Bâtiment Amphipôle, Lausanne, 1015, Switzerland; Department of Ecology and Evolution, University of Lausanne, Quartier Sorge, Bâtiment Biophore, Lausanne, 1015, Switzerland; Evolutionary Bioinformatics, SIB Swiss Institute of Bioinformatics, Quartier Sorge, Bâtiment Amphipôle, Lausanne, 1015, Switzerland; Department of Ecology and Evolution, University of Lausanne, Quartier Sorge, Bâtiment Biophore, Lausanne, 1015, Switzerland; Evolutionary Bioinformatics, SIB Swiss Institute of Bioinformatics, Quartier Sorge, Bâtiment Amphipôle, Lausanne, 1015, Switzerland; Department of Ecology and Evolution, University of Lausanne, Quartier Sorge, Bâtiment Biophore, Lausanne, 1015, Switzerland; Evolutionary Bioinformatics, SIB Swiss Institute of Bioinformatics, Quartier Sorge, Bâtiment Amphipôle, Lausanne, 1015, Switzerland; Department of Ecology and Evolution, University of Lausanne, Quartier Sorge, Bâtiment Biophore, Lausanne, 1015, Switzerland; Evolutionary Bioinformatics, SIB Swiss Institute of Bioinformatics, Quartier Sorge, Bâtiment Amphipôle, Lausanne, 1015, Switzerland; Department of Ecology and Evolution, University of Lausanne, Quartier Sorge, Bâtiment Biophore, Lausanne, 1015, Switzerland; Evolutionary Bioinformatics, SIB Swiss Institute of Bioinformatics, Quartier Sorge, Bâtiment Amphipôle, Lausanne, 1015, Switzerland; Department of Ecology and Evolution, University of Lausanne, Quartier Sorge, Bâtiment Biophore, Lausanne, 1015, Switzerland

## Abstract

Bgee (https://www.bgee.org/) is a database to retrieve and compare gene expression patterns in multiple animal species. Expression data are integrated and made comparable between species thanks to consistent data annotation and processing. In the past years, we have integrated single-cell RNA-sequencing expression data into Bgee through careful curation of public datasets in multiple species. We have fully integrated this new technology along with the wealth of other data existing in Bgee. As a result, Bgee can now provide one definitive answer all the way to the cell resolution about a gene’s expression pattern, comparable between species. We have updated our programmatic access tools to adapt to these changes accordingly. We have introduced a new web interface, providing detailed access to our annotations and expression data. It enables users to retrieve data, e.g. for specific organs, cell types or developmental stages, and leverages ontology reasoning to build powerful queries. Finally, we have expanded our species count from 29 to 52, emphasizing fish species critical for vertebrate genome studies, species of agronomic and veterinary importance and nonhuman primates.

## Graphical Abstract

**Graphical Abstract:**
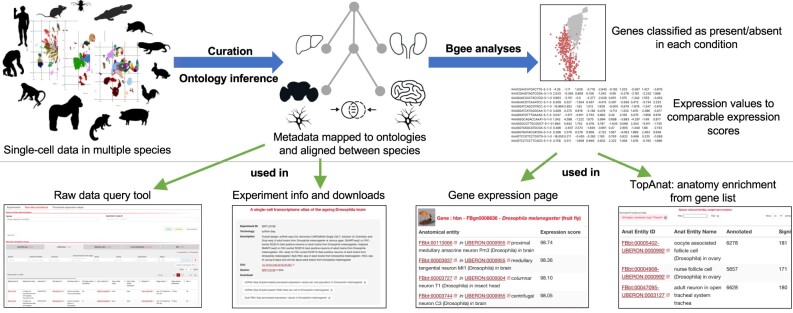

## Introduction

Bgee (https://www.bgee.org/) is a resource providing FAIR (Findable, Accessible, Interoperable and Reusable) (1) access to transcriptomics data, making gene expression information comparable between experiments and between species, and providing analysis tools to allow users to discover new information from their own data. Bgee focuses at integrating only healthy wild-type data to provide a clear reference of a gene expression pattern. Because of the usefulness of these data standardization and gene expression information, Bgee has been recognized in 2023 as a Global Core Biodata Resource (see https://globalbiodata.org/what-we-do/global-core-biodata-resources/) and as an Elixir Recommended Interoperability Resource (see https://elixir-europe.org/platforms/interoperability/rirs).

Since the publication of (2), we have focused our work on integrating single-cell RNA-sequencing (scRNA-seq) data, and improving data access by developing several new interfaces on the Bgee website, as well as improving programmatic access by updating our R packages, JSON API, download files and SPARQL endpoint.

### ScRNA-seq data integration

A first point of data integration for comparing expression data across experiments and species is the curation step, meaning, the capture of information about organs and cell types in a standardized way. Currently, most public datasets available are highly variable in the nomenclature used to name studied cell types, in part due to the complexity of classifying cells [see examples about neurons in (3), and about computational challenges in (4)]. Even the concept of cell populations as detected by current bioinformatics methods is elusive, and is confounded with cell states within a single population (5). Moreover, novel cell types are constantly being discovered, leading to challenges for standardizing their representation (6). This leads authors to often use their custom terms and abbreviations for referencing the fine-grained cell populations identified in their studies, which makes comparison of same populations across analyses highly challenging.

To circumvent these issues, large projects have a Data Coordination Platform to ensure annotations consistency across experiments, such as the Human Cell Atlas (7). But comparison between species is still difficult, even for homologous cell populations existing in multiple species, as projects can use different vocabularies, e.g. the Fly Cell Atlas (8) uses the FBbt cell type nomenclature (9), while the Human Cell Atlas uses the CL cell ontology (10). Other projects aim at consistently curating multiple experiments in multiple species by applying strict guidelines, such as CELLxGENE (11), enhancing comparisons and integration. But the scale of such efforts leads for instance CELLxGENE to focus at time of writing on human and mouse only.

Bgee is such a resource aiming at standardizing scRNA-seq datasets annotations across experiments and species for improving data reproducibility and integration. It also aims at making the resulting expression data comparable between experiments and species. This led notably Bgee and CELLxGENE to collaborate in the context of the consortium scFAIR (https://sc-fair.org/), to share annotation schema, best practices and dissemination.

### Rapid release of scRNA-seq annotations

Bgee supports researchers in three aspects: (i) providing an integrated view of a gene expression pattern, from all data available in Bgee, comparable between species; (ii) allowing the retrieval of standardized and annotated gene expression data; and (iii) providing analysis tools to make sense of users’ own data, such as TopAnat, a tool accepting a gene list as entry, to perform a test similar to a Gene Ontology (GO) (12) enrichment test, but testing instead the enrichment of anatomical terms, mapped to genes by expression patterns.

In the past, at each release of Bgee, all data annotated were analyzed and integrated in all the other tools, e.g. the gene page to retrieve a gene expression pattern, or TopAnat to perform enrichment analyses. With the rapid growth and lack of standardization of scRNA-seq data, it appeared that users needed a faster access to curated datasets, even before we could fully integrate them with all the other tools and data. For this reason, we now provide access through a new interface (see ‘Results’ section) to datasets that we have curated, but not yet integrated in the other tools. For instance, as of Bgee version 15.2, users can retrieve annotations for several experiments of the Human Cell Atlas, with analysis results that do not appear yet in the gene pages or in TopAnat. This will allow an increased reactivity of Bgee with regards to the curation of newly released scRNA-seq datasets. The datasets that have been curated will still be fully integrated at the following release of Bgee, to allow having more time for all the necessary analyses.

## Materials and methods

### Single-cell RNA-seq data integration

#### Multispecies cell type annotation

##### Ontologies and controlled vocabularies, and graph of conditions

For annotating expression data, Bgee annotates each sample or cell population to conditions that can capture information about different parameters: anatomy, developmental and life stage, sex, strain and physiological status. For each parameter, ontologies or controlled vocabularies are used: (i) for anatomy, the composite-metazoan version of Uberon (13), which merges into the multispecies anatomy ontology Uberon notably the cell ontology CL, and species-specific ontologies such as FBbt and ZFA (14); (ii) for developmental and life stages, the collection of ontologies available at https://github.com/obophenotype/developmental-stage-ontologies and merged within the structure of the minimal life stage ontology of Uberon; and (iii) for sex, strain and physiological status, controlled vocabularies are used, and for each controlled vocabulary all terms have one direct parent that is the root of the vocabulary (e.g. ‘any sex’ for sex, ‘wild type’ for strain since all data are healthy wild type in Bgee).

**Figure 1.**
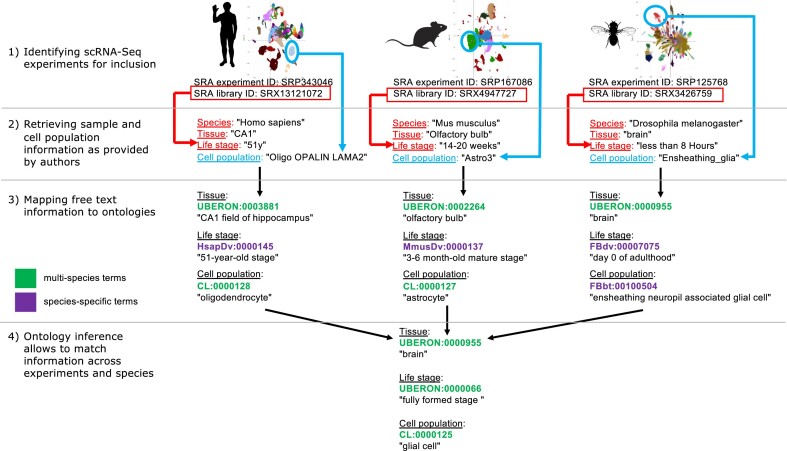
Workflow of the annotation and integration process of single-cell RNA-seq metadata in Bgee. (**1**) Experiments are targeted for inclusion in Bgee (real experiment and libraries IDs and real cell population annotations are provided, example clustering plots are used, real clustering plots from original studies not reproduced). (**2**) Sample and cell population information as provided by the authors in the original manuscript are retrieved. Tissue, developmental and life stage, sex (not shown) and strain (not shown) are annotated at the level of the library, as nonmultiplexed libraries contain one unique sample. Association from cell barcodes to cell clusters is retrieved, and each cell cluster is annotated to a cell type. (**3**) Curators map the free text information provided by authors to ontology terms. The composite version of Uberon is used, allowing to map to species-specific terms when necessary, and to multispecies terms when possible. (**4**) Annotations are propagated to all possible combinations of ancestral conditions in the ontologies, using post-composition. A user could for instance retrieve these single-cell data across species by querying all data for glial cells in adult brain.

Since Bgee relies on one single multispecies ontology, merging multiple resources into one, respectively, for anatomy and for development, it is possible to annotate data consistently with the same terms across species when they describe the same entity, or at least to infer a common parent across species when data are annotated with terms varying because of species biology (Figure 1). For instance, a cell population in experiment ERP129698 (8) in *Drosophila melanogaster* has been annotated to the term FBbt:00100482 ‘lobula columnar neuron Lcn10’; another cell population in experiment SRP222001 (15) in *Homo sapiens* has been annotated to the term CL:0000745 ‘retina horizontal cell’. These two different terms are reconnected within the structure of the composite-metazoan version of Uberon and are both considered subtypes of CL:0000099 ‘interneuron’. It is thus possible in Bgee to retrieve data for various types of interneurons across experiments and across species.

Each condition is then connected to other conditions using the relationships of the ontology or controlled vocabulary corresponding to each parameter, respectively. New parent conditions, not used in annotations, are also inferred using these relations. This allows creating a graph of conditions, allowing powerful queries, and expression data reconciliation (see below).

##### Anatomy and cell type post-composition

When capturing information about cell populations, the cell type is of course the main information to standardize, but the anatomical location (e.g. midbrain or hindbrain) is also crucial. One way to address this need is to use pre-composed terms already defined in the ontologies used. For instance, the term CL:0000082 ‘epithelial cell of lung’ is defined as the intersection of the terms CL:0000066 ‘epithelial cell’ and *part_of* UBERON:0000115 ‘lung epithelium’. If we were to create all possible combinations of terms used in annotations, this would lead to an unreasonable inflation of the number of terms in the ontology. It would also slow down the curation process, since many terms would have to be requested before being used in annotations.

To solve this issue, Bgee uses term post-composition. Information about anatomy is composed of the intersection of a cell type and of an anatomical structure, both represented individually in the composite-metazoan version of Uberon. This allows for more precise and faster curation of scRNA-seq datasets. While this is used internally for, e.g. reasoning to retrieve all conditions matching a query, this can be displayed conveniently to users as, e.g. two separate columns.

##### Original free text annotation retained

Since there is no standard definition of what is a cell population or a cell type, cell types can be described in studies with varying level of precision, for instance based on different sets of marker genes. For this reason, authors often use their own custom free text label to describe them. While mapping this free text information to an ontology term, some important information can be lost. For instance, the cell types used in experiment ERP111594 (16) ‘retinal bipolar neuron type A’ and ‘retinal bipolar neuron type B’ can only be mapped at time of writing to the same term CL:0000748 ‘retinal bipolar neuron’. Bgee always captures the free text annotation from authors in addition to ontology annotation, to allow users to get the maximum level of precision possible, while still allowing for standardization and retrieval of data using ontologies (Figure 1).

#### Comparable gene expression states and expression levels

There exists a variety of methods to try to produce an integrative analysis of multiple scRNA-seq datasets (17). A first step is to remove the batch effects due to, e.g. variation in protocols used. The challenge when integrating many datasets in a large database such as Bgee is that these methods can hardly be used automatically. For instance, why if a biological feature of interest is confounded with batch effect, e.g. all samples from one biological sex have been analyzed in one sequencing center, and samples from another biological sex in a different sequencing center? The biological signal of interest would be removed at the same time as the batch effect. This problem has been highlighted for instance using the mouse ENCODE data (18). In that paper, the authors identified that removing the confounding batch effect also removed most of the species effect on gene expression levels. The authors also noted that a similar case had been discussed in (19). For this reason, Bgee does not directly integrate gene expression count matrices between multiple experiments.

Instead, Bgee produces two types of information: qualitative present/absent expression calls, capturing the expression state of genes; and quantitative nonparametric ‘expression score’ metrics. These two types of information can then be combined between experiments of any data type (e.g. multiple scRNA-seq experiments, as well as bulk RNA-seq data). These metrics have been described in (2), and have now been applied to scRNA-seq data as well.

##### Present/absent expression calls

For present/absent expression calls (in preparation, preprint available at https://www.biorxiv.org/content/10.1101/2022.03.31.486555v1, DOI 10.1101/2022.03.31.486555), briefly: first, to determine the expression state of genes, we identify a stringent set of intergenic regions, free of unannotated genes. For this, we use bulk RNA-seq data for each species, to detect dubious intergenic regions based on their expression level. Second, we use these stringent sets of intergenic regions to define the background level of expression noise in each sample: from the distribution of intergenic expression values, we produce for each gene in each sample a *P*-value against the null hypothesis that it belongs to the same distribution. Third, we integrate for each gene all *P*-values from all data available in a condition and its child conditions, using our graph of conditions (see above). As a result, we produce one *P*-value per gene and condition: if the *P*-value is significant, the gene is classified as having a ‘present’ expression call in that condition, otherwise, an ‘absent’ expression call.

In the case of scRNA-seq data, to obtain enough signal, we pseudo-bulk the data from all cells belonging to a same population in a library, meaning that we aggregate all reads from the individual cells to study the population. To define cell populations, we use the original annotations as provided by the authors of a study in the reference paper, we do not re-cluster the data ourselves. The method then behaves as for bulk RNA-seq data.

##### Expression score

We also produce a nonparametric quantitative score related to the expression level of genes, going from 0 to 100, 100 representing the top expressed gene in a condition. Also described in (2), briefly: first, we rank genes in a sample based on their expression level. Second, we normalize these ranks, to account for the fact that not all gene expression levels can be determined, depending on the technology or protocol used. For instance, some protocols capture only messenger RNAs (mRNAs) with a poly-A tail, while some protocols capture all mRNAs and then perform a ribo-depletion. Third, we integrate all normalized ranks for a gene from all available data in a condition and its child conditions, using our graph of conditions (see above). Finally, we invert the ranks and renormalize to obtain an expression score between 0 and 100 for each gene and each condition. In the case of scRNA-seq data, we pseudo-bulk the data as described above.

### Integration of new species

Since the publication of (2), Bgee includes 23 more species, emphasizing fish species critical for vertebrate genome studies, species of agronomic and veterinary importance and nonhuman primates (see https://www.bgee.org/search/species for the most recent list of species included in Bgee). We retrieved genome annotations from either Ensembl (20), EnsemblMetazoa (21) or Refseq (22). We retrieved RNA-seq data from the Sequence Read Archive (SRA) (23). We updated the ontologies used to capture conditions (see above), notably to recompute the taxon constraints (24) for these new species, meaning, computing which term exists for each species. We have applied our analyses to produce present/absent expression calls and expression scores (see above).

### Development of new query tools

We have released a new web interface with new query tools, and a new JSON API. For storing data, we use MySQL (https://www.mysql.com/). We develop our backend with the Java language (https://www.java.com/), using the open-source implementation from OpenJDK (https://openjdk.org/). We develop our frontend with the React framework (https://react.dev/). We serialize our Java classes into JSON using the Gson library (https://github.com/google/gson). The source code of our backend is available at https://github.com/BgeeDB/bgee_apps/ under the license CC0. The source code of our frontend is available at https://github.com/BgeeDB/bgee-unil under the license CC0. See the repositories to see which versions of tools are used.

We also provide data access and computations through packages developed in R (https://www.r-project.org/). The source code of our package BgeeDB (25) is available at https://github.com/BgeeDB/BgeeDB_R under the license GPL-3.0. The source code of our package BgeeCall (https://bioconductor.org/packages/BgeeCall/) to produce our present/absent expression calls is available at https://github.com/BgeeDB/BgeeCall under the license GPL-3.0.

## Results

### Retrieving curated datasets through a powerful query tool

**Figure 2.**
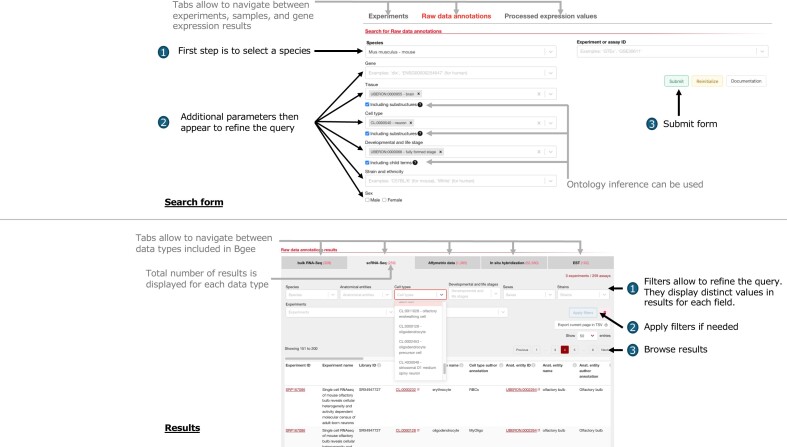
Depiction of the interface to query data annotated in Bgee available at https://www.bgee.org/search/raw-data. Top panel renders the search form with steps to use. Bottom panel renders the search results with steps to refine query.

We developed a new interface (Figure 2) allowing to retrieve experiments (https://www.bgee.org/search/raw-data), samples or cell populations (https://www.bgee.org/search/raw-data?pageType=raw_data_annots) and gene read counts (https://www.bgee.org/search/raw-data?pageType=proc_expr_values), based on several parameters, notably using ontology reasoning. The first step is to select either a species or an experiment. Once a species is selected, the other parameters appear, with an autocompletion feature.

- For genes, either the gene name or the gene ID can be used. Several genes can be selected, acting as an OR operator.
- For tissues, cell types and developmental and life stages, either the name or the ID of the terms can be used. For each parameter, several terms can be selected, acting as an OR operator. A checkbox selects for retrieval of annotations mapped to any child term of the selected terms, using the relationships from the respective ontologies. For instance, when selecting the term CL:0000099 ‘interneuron’, and checking this checkbox, in human, three cellular subtypes used in annotations (as of Bgee 15.2) are retrieved: CL:0000561 ‘amacrine cell’, CL:0000745 ‘retina horizontal cell’ and CL:0000748 ‘retinal bipolar neuron’.
- For ‘strain and ethnicity’, and for ‘sex’, a controlled vocabulary is used, with no relations between terms beside the root of the vocabulary. There is no checkbox to select child terms.
- The checkbox ‘Data integration’ allows to select the datasets that are fully integrated in all the other tools of Bgee, and that are not at the time released as curated datasets only (see ‘Introduction’ section).

Above the results, several filters are displayed to refine user’s query. These filters display the actual values that are observed in the retrieved datasets. For instance, when a user performs a query for the term CL:0000099 ‘interneuron’ in human, including all its child terms, the filter ‘Cell types’ display the three terms (as of Bgee 15.2) listed above that are observed in the retrieved datasets satisfying the query.

### Experiment page

A new page has been developed to retrieve information and download files related to a specific experiment (e.g. https://www.bgee.org/experiment/ERP111594 for experiment ERP111594). This page lists all sample or cell population annotations, as well as other useful information, such as experiment description, technology used, DOI of the associated paper and link to source database hosting the raw data.

Several download files are available. For instance, for scRNA-seq experiments using droplet-based technologies (e.g. 10X Genomics), one TSV file allows to retrieve the read counts per gene from the pseudo-bulk of each cell population, and a H5AD file allows to retrieve the gene read counts per cell.

Bgee does not provide Uniform Manifold Approximation and Projection (UMAP) or t-distributed stochastic neighbor embedding (t-SNE) plot visualizations related to an experiment, along with, e.g. annotations and gene expression values. The reason is that performing a fully automated cell clustering would not reproduce the clustering as performed by the authors of the study, that is most of the time fine-tuned. Coordinates of each cell in the dimension reduction plot is almost never deposited along with the raw data in primary repositories. Deposition-based resources such as CELLxGENE make mandatory to deposit these coordinates at time of submission, but Bgee curates primary repositories and is not deposition-based.

### Gene expression patterns, from organs to cell resolution

Our gene page displays a gene’s expression pattern from the integration of all data available in Bgee. Expression locations are ordered based on our expression score, highlighting first the domains of expression with the highest levels of expression. ScRNA-seq data are integrated along with all other data types present in Bgee (e.g. bulk RNA-seq data, *in situ* hybridization data). As a result, users can retrieve gene expression domains from the organ level up to the cell resolution.

For instance, in the case of the gene FBgn0008636 [FlyBase (26) identifier] ‘hbn’ in *D. melanogaster* (https://www.bgee.org/gene/FBgn0008636, archived version Bgee 15.2 https://www.bgee.org/bgee15-2/gene/FBgn0008636), the top four terms are specific neuron types showing the highest levels of expression, coming from scRNA-seq data. The 17th entry, expression in FBbt:01000119 ‘anterior ectoderm anlage’, has been observed by *in situ* hybridization data. Expression in UBERON:6003007 ‘insect adult head’ has been shown using different data types: bulk RNA-seq, scRNA-seq and *in situ* hybridization.

Of note, these examples come from retrieving expression domains in anatomy only, but Bgee allows retrieving information for any combination of the condition parameters: anatomy, developmental and life stage, sex and strain. Users can also restrict the results to some technologies, e.g. retrieving results from scRNA-seq data only. For each result, the link ‘See source data’ allows to identify precisely the source data that were used to produce that result.

### Gene expression enrichment up to the cellular expression level

TopAnat (https://www.bgee.org/analysis/top-anat/) is a tool provided by Bgee allowing performing GO-like enrichment of anatomical terms, mapped to genes using our present/absent expression calls. It accepts as entry a list of genes, and provides as result the anatomical domains where that list of genes is significantly more often expressed as compared with a background list of genes (all genes in the genome by default).

With the integration of scRNA-seq data, TopAnat can now provide this information at the cellular resolution, i.e. the specific cell populations where the expression of the list of genes is enriched. For instance, when considering all genes in *D. melanogaster* associated to the phenotype ‘reduced fertility’, it is possible to discover the precise cell populations where these genes have an enriched expression (see https://www.bgee.org/bgee15-2/analysis/top-anat/55574711b6b7916fddcff5af83ce10200b88cb3f for results for archived version Bgee 15.2). The top two terms are FBbt:00005402 ‘oocyte associated follicle cell’ and FBbt:00004908 ‘nurse follicle cell’, both in UBERON:0000992 ‘ovary’.

### Expression call query tool

We also released an interface allowing retrieving our present/absent expression calls, along with expression scores, as displayed on the gene pages, but with enhanced query capabilities (https://www.bgee.org/search/expression-calls). Users must first select a species, then one or several genes, thanks to an autocompletion field accepting gene IDs or gene names. As in the interface allowing to retrieve curated datasets (see above), results can be filtered by tissue, cell type, developmental and life stage, sex and strain, using ontology reasoning when appropriate. Additional parameters are provided:

- Data type: users can restrict the data types used to produce the results, e.g. to retrieve results produced by scRNA-seq data only.
- Condition parameters: users can retrieve present/absent expression calls for any combination of the condition parameters (anatomy, developmental and life stage, sex and strain).
- Call type: users can choose to retrieve any present/absent expression calls, or only present calls or only absent calls.
- Data quality: users can restrict the minimum level of confidence in the gene expression result.

### Programmatic access

To allow programmatic access to Bgee data, we provide download files, R packages, a JSON API and a SPARQL endpoint. They have been updated to allow access to single-cell data. Files containing present/absent expression calls can be retrieved per species at https://www.bgee.org/download/gene-expression-calls. New columns allow retrieving information about scRNA-seq data. Processed expression values can be retrieved per species at https://www.bgee.org/download/processed-expression-values. New files are provided for scRNA-seq technologies: TSV files containing information about experiment and libraries; TSV files containing read count information per cell population; and H5AD files containing read count information per cell.

Our package BgeeDB (https://bioconductor.org/packages/BgeeDB/) to access our annotations and processed expression values has been updated to support scRNA-seq technologies. Our package BgeeCall (https://bioconductor.org/packages/BgeeCall/) has been updated allowing computing present/absent expression calls for scRNA-seq technologies.

We have updated our SPARQL endpoint to support single-cell data, notably annotation of cell type in the knowledge graph, see https://www.bgee.org/sparql/.

We have released a new JSON API allowing access to all our data, with documentation available at https://www.bgee.org/doc-api/.

### New species integrated, focus on nonmodel organisms

Bgee includes 23 more species as compared with the publication of (2). While Bgee includes the classical model organisms (human, mouse, fly, zebrafish and *C. elegans*), it also supports researchers working on nonmodel organisms. For instance, Bgee has collaborated with the SalmoBase project (27) to integrate all healthy wild-type data available in SRA at time of writing for the species *Salmo salar*. These data are now available in all our tools, and the curated datasets can be retrieved with our new interface described above.

## Discussion

Single-cell genomics data are challenging regarding data FAIRification, as new technologies are constantly being developed (see https://www.bgee.org/support/scRNA-seq-protocols-comparison for a comparative guide of scRNA-seq protocols), and the parameters of analysis pipelines are highly variable, leading to vastly different results to define what cell populations are. The approach used by Bgee is pragmatic: we trust the authors of a study to best know their data and the analysis results, and we aim at standardizing their own definition of the cell populations they identified.

We directed our efforts toward defining how to represent such data in a FAIR manner, and adapting our analyses for present/absent calls and expression scores to single-cell data. This represented several years of work and benchmarking. Notably, most methods do not evaluate their results in nonmodel organisms. Our methods yield comparable results in model and nonmodel species. We are happy to present here the updated version of Bgee, where single-cell data are fully integrated with all tools and existing data, and applicable to all species included. We also streamlined the release of our curation of single-cell datasets for improved reactivity and for better service to our users.

In the upcoming releases of Bgee, we will focus on scaling up new dataset integration, notably for a greater diversity of nonmodel species.

## Data Availability

All data provided by Bgee are under the permissive license CC0. Bgee provides archived version with permanent URLs up to the 2016 release. All source code of Bgee is public and either under license CC0 or GPL3. All original data used in Bgee are listed, with links to the original repositories hosting them (e.g. SRA). All source code of tools mentioned in the present article are archived on Figshare under DOIs: 10.6084/m9.figshare.27022375, 10.6084/m9.figshare.27022372, 10.6084/m9.figshare.27022366, 10.6084/m9.figshare.27022369 and 10.6084/m9.figshare.27022363.

## Notes

Present address: Wan-Ting Huang, Department of Computational Biology, University of Lausanne, Lausanne, 1015, Switzerland.

Present address: Marianna Tzivanopoulou, University Grenoble Alpes, France.
